# HM_ADET: a hybrid model for automatic detection of eyelid tumors based on photographic images

**DOI:** 10.1186/s12938-024-01221-3

**Published:** 2024-02-28

**Authors:** Jiewei Jiang, Haiyang Liu, Lang He, Mengjie Pei, Tongtong Lin, Hailong Yang, Junhua Yang, Jiamin Gong, Xumeng Wei, Mingmin Zhu, Guohai Wu, Zhongwen Li

**Affiliations:** 1 School of Electronic Engineering, Xi’an University of Posts and Telecommunications, Xi’an, 710121 China; 2 School of Computer Science and Technology, Xi’an University of Posts and Telecommunications, Xi’an, 710121 China; 3 School of Modern Post, Xi’an University of Posts and Telecommunications, Xi’an, 710061 China; 4 School of Communications and Information Engineering, Xi’an University of Posts and Telecommunications, Xi’an, 710121 China; 5 School of Mathematics and Statistics, Xidian University, Xi’an, 710071 China; 6 Ningbo Eye Hospital, Wenzhou Medical University, Ningbo, 315000 China; 7 School of Ophthalmology and Optometry and Eye Hospital, Wenzhou Medical University, Wenzhou, 325027 China

**Keywords:** Object localization, YOLOv7, Eyelid tumors, Malignancy, Vision transformer

## Abstract

**Background:**

The accurate detection of eyelid tumors is essential for effective treatment, but it can be challenging due to small and unevenly distributed lesions surrounded by irrelevant noise. Moreover, early symptoms of eyelid tumors are atypical, and some categories of eyelid tumors exhibit similar color and texture features, making it difficult to distinguish between benign and malignant eyelid tumors, particularly for ophthalmologists with limited clinical experience.

**Methods:**

We propose a hybrid model, HM_ADET, for automatic detection of eyelid tumors, including YOLOv7_CNFG to locate eyelid tumors and vision transformer (ViT) to classify benign and malignant eyelid tumors. First, the ConvNeXt module with an inverted bottleneck layer in the backbone of YOLOv7_CNFG is employed to prevent information loss of small eyelid tumors. Then, the flexible rectified linear unit (FReLU) is applied to capture multi-scale features such as texture, edge, and shape, thereby improving the localization accuracy of eyelid tumors. In addition, considering the geometric center and area difference between the predicted box (PB) and the ground truth box (GT), the GIoU_loss was utilized to handle cases of eyelid tumors with varying shapes and irregular boundaries. Finally, the multi-head attention (MHA) module is applied in ViT to extract discriminative features of eyelid tumors for benign and malignant classification.

**Results:**

Experimental results demonstrate that the HM_ADET model achieves excellent performance in the detection of eyelid tumors. In specific, YOLOv7_CNFG outperforms YOLOv7, with AP increasing from 0.763 to 0.893 on the internal test set and from 0.647 to 0.765 on the external test set. ViT achieves AUCs of 0.945 (95% CI 0.894-0.981) and 0.915 (95% CI 0.860-0.955) for the classification of benign and malignant tumors on the internal and external test sets, respectively.

**Conclusions:**

Our study provides a promising strategy for the automatic diagnosis of eyelid tumors, which could potentially improve patient outcomes and reduce healthcare costs.

**Supplementary Information:**

The online version contains supplementary material available at 10.1186/s12938-024-01221-3.

## Background

Eyelid tumors are the most commonly encountered neoplasm in routine ophthalmology clinics, which can be categorized into benign and malignant based on their pathogenesis [1]. Due to their proximity to vital organs such as the eyeballs and brain, eyelid tumors pose a great threat to the normal functions of these organs. Malignant tumors, in particular, are prone to invasion and metastasis, resulting in blindness, disability, and even death [2, 3]. The estimated survival rate of malignant eyelid tumors can reach a rate of 99% over 5 years if they could be located and treated in the earliest stage [4]. However, the large-scale screening of eyelid tumors is limited by the sparse and uneven distribution of experienced ophthalmologists [5, 6]. As a result, many suspicious patients may not be accurately diagnosed in a timely manner. To bridge the gap in manual diagnosis defects in ophthalmology, it is imperative to develop an automated diagnosis system for eyelid tumors.

With the accumulation of medical images and the development of artificial intelligence, deep learning (DL) algorithms have achieved unprecedented performance in the automatic diagnosis and lesion localization of various ophthalmic diseases, including eyelid tumors [7, 8], keratitis [9], cataract [10, 11], glaucoma [12, 13], and diabetic retinopathy (DR) [14, 15]. Among them, automatic diagnosis of eyelid tumors has received widespread attention from scholars and medical professionals due to their life-threatening potential and increasing frequency of incidence. Andayni et al. applied a backpropagation neural network for the automatic identification of retinoblastoma using fundus images, with an accuracy of 90% [16]. Oyebade et al. employed convolutional neural networks (CNNs) and deep belief networks (DBNs) to diagnose iris nevus automatically, achieving exceptional accuracies of 93.35% and 93.67%, respectively [17]. Jaya et al. applied an extreme learning machine (ELM) for the automatic diagnosis of retinoblastoma with an accuracy of 92% [18]. Adamopoulos et al. utilized multi-layer error backpropagation and CNNs to classify eyelid basal cell carcinomas and healthy individuals based on 143 full-face or half-face images derived from a single clinical center, achieving an accuracy of 80% [7]. Li et al. developed an artificial intelligence algorithm for the diagnosis of benign and malignant eyelid tumors with an accuracy of 82.2% [8]. Hui et al. used ResNet101 to identify benign and malignant eyelid tumors based on 36 clinical images, with an accuracy of 88.9% [19].

Although the aforementioned studies have demonstrated the potential of artificial intelligence and machine learning algorithms to automatic diagnosis of eyelid tumors, their performance is still far inferior to that of experienced ophthalmologists, making it difficult to be applied to real-world clinical practice. This is mainly due to the fact that eyelid tumors, as a type of tumor, have their own unique features on photographic images compared to other eye diseases. Eyelid tumors have the characteristics of small lesions and uneven distribution in the early stage of onset, and the lesions are surrounded by a large amount of irrelevant noise. If the original images are adopted directly, redundant noise will inevitably be extracted and transmitted to the downstream classifier, ultimately affecting the accuracy of the final decision. Therefore, it is essential to explore an automatic method to locate eyelid tumors prior to diagnosis. Furthermore, early-stage eyelid tumors are usually atypical, with numerous similar color and texture features observed across different categories, and some variations exhibited within the same category. These factors pose a high challenge for achieving accurate diagnosis of eyelid tumors.

In this study, a hybrid model HM_ADET for automatic detection of eyelid tumors was proposed, including an automatic localization algorithm YOLOv7_CNFG to locate eyelid tumors and vision transformer (ViT) to classify benign and malignant eyelid tumors. In specific, the ConvNeXt module with an inverted bottleneck layer was employed to avoid information loss of small eyelid tumors during the downsampling process. The flexible rectified linear unit (FReLU) activation function, specifically designed for visual tasks, maps the output of the neural network into a nonlinear space. This nonlinear mapping can capture the multi-scale features, such as texture, edge, and shape, making it more effective in classifying small eyelid tumors. The generalized intersection over union loss (GIoU_loss) can effectively measure the geometric difference between the predicted box (PB) and the ground truth box (GT), guiding the network to optimize the location, size, and shape of objects, and improve the localization accuracy of eyelid tumors. Second, to address the challenge of identifying atypical features in early-stage eyelid tumors, we explored the efficacy of six different convolutional neural networks, including AlexNet [20], VGG19 [21], Inception-v3 [22], ResNet101 [23], DenseNet121 [24], and ViT [25], applied to cropped tumor regions, to determine the most suitable classifier for eyelid tumor diagnosis. A multi-head attention (MHA) module was incorporated in the ViT to extract tumor features from multiple perspectives, and then merge them to obtain a more comprehensive feature representation. This technique enabled the ViT to focus on subtle differences among similar images, which is beneficial for accurately classifying benign and malignant eyelid tumors. Finally, detailed qualitative and quantitative experiments were conducted to determine the optimal diagnosis model for eyelid tumors, and the YOLOv7_CNFG combined with ViT algorithm achieved the best performance on the internal and external test sets, with accuracy rates of 92.1% (95% CI 88.2–95.9) and 92.8% (95% CI 89.8–95.9), respectively.

To sum up, the main contributions of this work can be list below.

1. A hybrid model HM_ADET is proposed for automatic detection of eyelid tumors, which includes YOLOv7_CNFG to locate eyelid tumors and ViT to classify benign and malignant eyelid tumors.
2. The ConvNeXt, FReLU, and GIoU_loss techniques are integrated into YOLOv7 model to enhance the localization accuracy of eyelid tumors, especially small targets.
3. The effectiveness of six different convolutional neural networks is investigated to address the challenge of identifying atypical features of eyelid tumors, and the most suitable classifier for eyelid tumor diagnosis is determined.
4. Extensive experiments are conducted on the internal and external eyelid tumor datasets, validating that the HM_ADET achieves excellent performance in the automatic detection of benign and malignant eyelid tumors.

## Results

### Datasets

#### Acquisition of materials

In this study, a total of 1151 photographic images with 1248 eyelid tumors were collected from Ningbo Eye Hospital (NEH) using ordinary digital cameras between January 2010 and March 2021. The NEH dataset was utilized to develop and validate the performance of the localization and classification methods of eyelid tumors. To further confirm the effectiveness and generalization ability of the deep learning algorithms, we also collected and annotated an additional dataset of 266 photographic images from two other ophthalmology hospitals. This external dataset comprised 215 photographic images collected from Jiangdong Eye Hospital (JEH) and 51 photographic images collected from Zunyi First People’s Hospital (ZFPH).

Two junior ophthalmologists with 2-year clinical experience were recruited to annotate the cropped images. Prior to the annotation of eyelid tumor boundaries by junior ophthalmologists, they received standardized training under the guidance of experienced ophthalmologists. In specific, two junior ophthalmologists initially annotated the boundaries of the eyelid tumors, followed by a review and verification of the annotations by an experienced ophthalmologist. Corrections were only made for a few eyelid tumor samples with unclear boundaries. The main implementation by junior ophthalmologists, along with the guidance and inspection by experienced ophthalmologists, ensured both data quality and annotation efficiency. The label of each cropped image was determined based on an unequivocal histopathological diagnosis. The images of the development and the external test sets were taken at different locations, including outpatient clinics, hospital wards, and operating rooms, resulting in uneven backgrounds and lighting conditions, demonstrating the diversity of our dataset. The dataset was randomly divided into three subsets with a ratio of 0.7:0.15:0.15 for training, validation, and testing, respectively.

The dataset used for automatic localization of eyelid tumor includes a training set of 805 images, a validation set of 173 images, a test set of 173 images, and an external test set of 266 images. As each image potentially contains one or more lesion regions, the dataset cropped by YOLOv7_CNFG for the automatic classification of benign and malignant eyelid tumors includes an internal dataset of 1248 image patches and an external dataset of 279 image patches. The detailed distribution of benign and malignant eyelid tumors is presented in Table 1. Four types of benign eyelid tumors (BET) are included in the dataset, namely pimented naevus (PN), seborrheic keratosis (SK), squamous cell papilloma (SCP), and other benign tumor (OBT). Malignant eyelid tumors (MET) are also classified into four types, including sebaceous carcinoma (SC), basal cell carcinoma (BCC), squamous cell carcinoma (SCC), and other malignant tumor (OMT).

**Table 1:** Distribution of benign and malignant eyelid tumors

Type	Internal dataset				External dataset
	Train	Val	Test	Total	Test
BET	672	132	139	943	222
MET	200	55	50	305	57
Total	872	187	189	1248	279

Internal dataset from NEH, external dataset from JEH and ZFPH

#### Data pre-processing

**Fig. 1:**
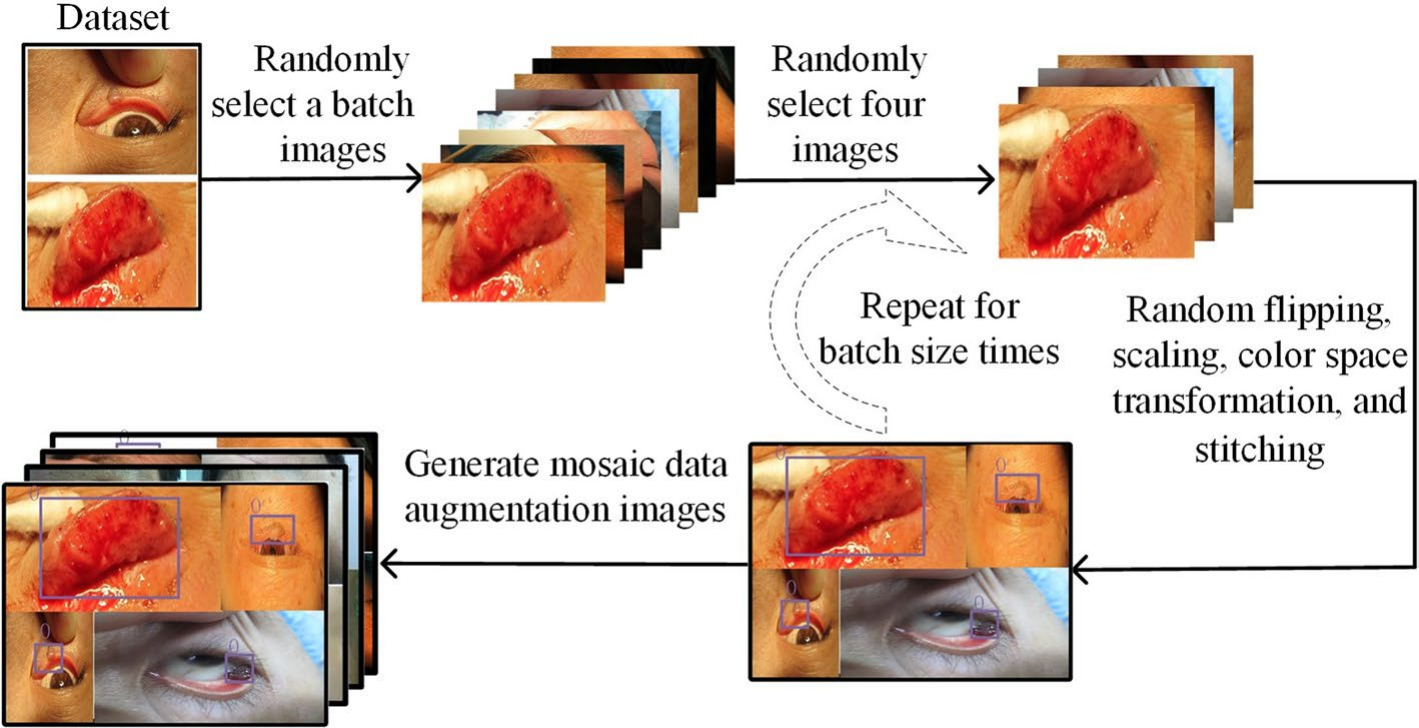
The implementation process of mosaic data augmentation

Medical images are often susceptible to noise and imaging quality. To increase the diversity of the dataset, different techniques of data augmentation, such as random rotation, horizontal and vertical flipping, and brightness adjustments are applied to the training dataset. In addition, mosaic data augmentation is employed to further enhance the model’s generalization ability and prevent overfitting problem [26]. Figure 1 shows the implementation process of the mosaic data augmentation process. First, a batch of images is randomly selected from the eyelid tumor dataset. Then, from this batch, four images are chosen at random and subjected to horizontal flipping, scaling, and color space transformation. These four transformed images are then concatenated together to form a new image. After repeating this operation for batch sizes times, the generated images are fed into the target localization network for training. The mosaic data augmentation technique significantly improves the robustness of the model by combining semantic information from four images [27].

#### Evaluation metrics and statistical analysis

The primary metrics used to evaluate the performance of eyelid tumors localization are precision, recall, average precision (AP), and precision–recall (PR) curves. These metrics are calculated using Eqs. (1–3).

1 $$Precision{ = }\frac{TP}{{TP + FP}}$$

2 $$Recall{ = }\frac{TP}{{TP + FN}}$$

3 $$AP = \int_{0}^{1} {P{(}R{)}dR}$$

where TP (True Positive) represents the number of correctly located eyelid tumor regions. FP (False Positive) indicates the number of incorrectly located eyelid tumor regions, and FN (False Negative) indicates the number of missed eyelid tumor regions. Precision is the ratio of correctly located eyelid tumor regions to the total number of located eyelid tumor regions, while recall is the ratio of correctly located eyelid tumor regions to the total number of eyelid tumor regions in the dataset.

Evaluation metrics used for the automatic classification of eyelid tumors include sensitivity, specificity, accuracy, F1-score (F1), receiver operating characteristic (ROC) curve, area under the ROC curve (AUC), and confusion matrix. The ROC is a particularly important evaluation metric that illustrates the relationship between sensitivity (true positive rate) and 1-specificity (false positive rate). F1 is calculated using the formula (4).

4 $$F1 = \frac{2*TP}{{2TP + FP + FN}}$$

where TP, FP, and FN denote the number of true positives, false positives, and false negatives in the classification results, respectively. All models were evaluated using the one-versus-rest strategy. Statistical analyses were performed with Python 3.7.8 and the Scikit-learn package. The Wilson Score Approach was used to calculate the 95% confidence intervals (CI) for accuracy, specificity, and sensitivity, while Empirical Bootstrap with 2000 iterations was utilized to calculate the 95% CI for AUC.

#### Experimental setup

Extensive experiments were conducted on an Ubuntu18.04 64-bit operating system using four NVIDIA TITAN RTX GPUs with CUDA 10.2, CUDNN 7.6.5, Pytorch 1.7.0, and Python 3.7.8. For eyelid tumors localization, the number of iterations (epoch) was set to 300. In the classification experiment, the size of mini-batch was set to 32 on each GPU to expedite the convergence of model parameters, and the initial learning rate and the maximum epoch were set to 1e-03 and 30, respectively. The learning rate was successively reduced to one-tenth of the original value in steps of 10 epoch. The model achieving the highest validation accuracy was chosen for use on the internal test set.

### Experimental results

#### Performance comparison of different localization algorithms for eyelid tumors

To evaluate the effectiveness of the proposed YOLOv7_CNFG model in locating eyelid tumor, we selected YOLOv7, YOLOv7 + ConvNeXt, and YOLOv7 + ConvNeXt + FReLU as comparative models. The precision, recall, and AP of these four localization models are statistically presented in Table 2. The YOLOv7_CNFG model outperformed the other three models with an AP of 0.893 and a recall of 0.906 on the internal test set, as well as an AP of 0.765 and a recall of 0.800 on the external test set. Meanwhile, the YOLOv7 + ConvNeXt achieved the highest precision of 0.918 and 0.847 on the internal and external test sets, respectively. Notably, compared to the YOLOv7 model, the AP of the YOLOv7_CNFG model had improved by 0.13 and 0.118 on the internal and external test sets, respectively.

**Table 2:** Performance comparison of different localization models for eyelid tumors

Model	Internal test set			External test set		
	Precision	Recall	AP	Precision	Recall	AP
YOLOv7	0.907	0.772	0.763	0.777	0.745	0.647
YOLOv7 + ConvNeXt	**0.918**	0.795	0.796	**0.847**	0.724	0.692
YOLOv7 + ConvNeXt + FReLU	0.878	0.850	0.836	0.825	0.782	0.745
YOLOv7_CNFG	0.898	**0.906**	**0.893**	0.844	**0.800**	**0.765**

The bold font represents the optimal performance in a column

To further demonstrate the performance of YOLOv7_CNFG, we conducted an extensive comparison between YOLOv7_CNFG and various YOLOx models with different network structures, including model size, trainable parameters, running time of testing, giga floating-point operations per second (GFLOPs), and AP. As shown in Table 3, although YOLOv7_CNFG fell behind YOLOx-s in terms of model size, parameters, and GFLOPs, it outperformed YOLOx-s by achieving faster testing time and higher AP in eyelid tumor detection. Notably, the AP of the YOLOv7_CNFG reached an impressive 0.893, which can be attributed to three key factors. First, the employed re-parameterization technique serves as a post-training enhancement method, which extends the training process and yields enhanced inference outcomes. Second, the FReLU activation function adeptly captures multi-scale features of eyelid tumors. Last, the GIoU_Loss proves effective in accurately locating eyelid tumors with diverse shapes and irregular boundaries. Moreover, the testing time of the YOLOv7_CNFG (only 0.191 s) exceeded that of YOLOx, primarily due to the utilization of a composite model scaling technique within YOLOv7_CNFG. This strategy enhanced its adaptability to various computing devices, thus meeting higher speed requirements.

**Table 3:** Comparative analysis of efficiency and AP between YOLOv7_CNFG and YOLOx models

Model	Size (MB)	Parameters (M)	Testing time (s)	GFLOPs	AP
YOLOv7_CNFG	71.3	36.5	**0.191**	103.20	**0.893**
YOLOx-s	**68.5**	**18.4**	0.255	**28.70**	0.780
YOLOx-m	193.0	25.3	0.338	73.73	0.786
YOLOx-l	413.0	54.2	0.534	155.67	0.809
YOLOx-x	756.0	99.0	0.931	282.03	0.812

Testing time indicates the average time that the method needs in testing one photographic image. *MB* Mbyte

**Fig. 2:**
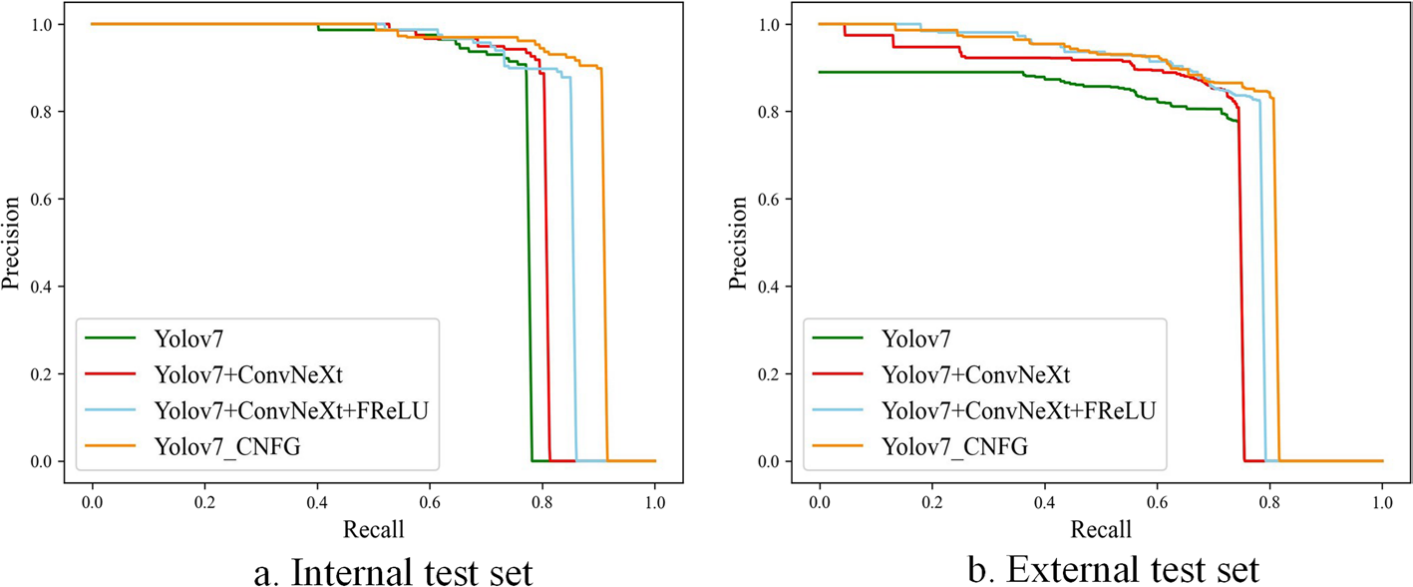
PR curves of YOLOv7_CNFG and the compared models for eyelid tumors localization. **a** PR curves on the internal test set. **b** PR curves on the external test set. *PR* precision–recall

Figure 2 illustrates the PR curves of the four models. In Fig. 2a, the precision of YOLOv7_CNFG showed a slight decrease when the recall was greater than 0.5, while the precision of other methods began to decrease rapidly when the recall was greater than 0.8. In Fig. 2b, the YOLOv7_CNFG model consistently maintained the highest precision when the recall was larger than 0.3. The YOLOv7_CNFG was closest to the upper right corner, indicating its satisfactory performance in locating eyelid tumors.

**Fig. 3:**
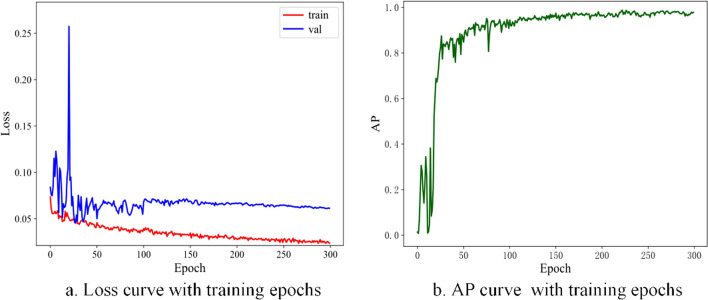
Loss and AP curves of the YOLOv7_CNFG model with epochs. *AP* average precision, Epoch the number of iterations

Figure 3 presents the loss and AP curves over 300 epochs. In Fig. 3a, the loss of training dataset gradually decreased with increasing epochs, indicating the progressive optimization of the model. After 200 epochs, the loss approached a minimal value. The loss of validation dataset initially exhibited fluctuates, eventually stabilizing after 100 epochs, demonstrating the model’s reasonable performance. In Fig. 3b, initially, the model systematically familiarized itself with the underlying data characteristics, optimizing parameters for a rapid ascent in AP. As the number of epochs increased, AP stabilized consistently within the favorable range of 0.9–1. The trends observed in these curves further verified the effectiveness and convergence of the proposed model.

**Fig. 4:**
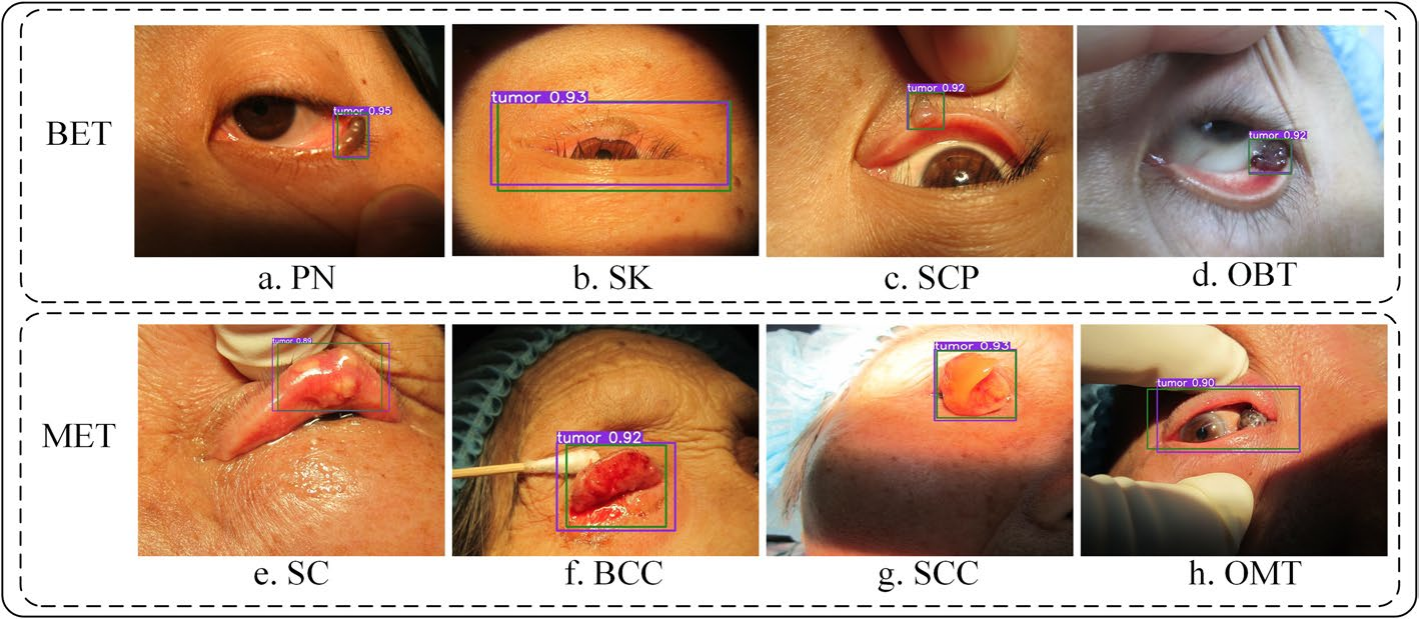
Representative localization results for benign and malignant eyelid tumors using YOLOv7_CNFG. The green box refers to the ground truth of eyelid tumors. The purple box refers to the localization result using YOLOv7_CNFG. The purple numerical values indicate confidence scores (range 0–1) which reflect the degree of confidence that the prediction box covers an eyelid tumor. *PN* pimented naevus, *SK* seborrheic keratosis, *SCP* squamous cell papilloma, *OBT* other benign tumor, *SC* sebaceous carcinoma, *BCC* basal cell carcinoma, *SCC* squamous cell carcinoma, *OMT* other malignant tumor

To visually evaluate the performance of YOLOv7_CNFG in locating eyelid tumors, Fig. 4 displays the localization results of several representative cases. The ground truth boxes are depicted in green, while the purple boxes illustrate the localization results of YOLOv7_CNFG. The experimental results clearly indicated that the automatic localization boxes generated by the YOLOv7_CNFG model were remarkably consistent with the expert-annotated boxes, highlighting its impressive ability to locate eyelid tumors.

#### Performance comparison of the automatic diagnosis of benign and malignant eyelid tumors

**Fig. 5:**
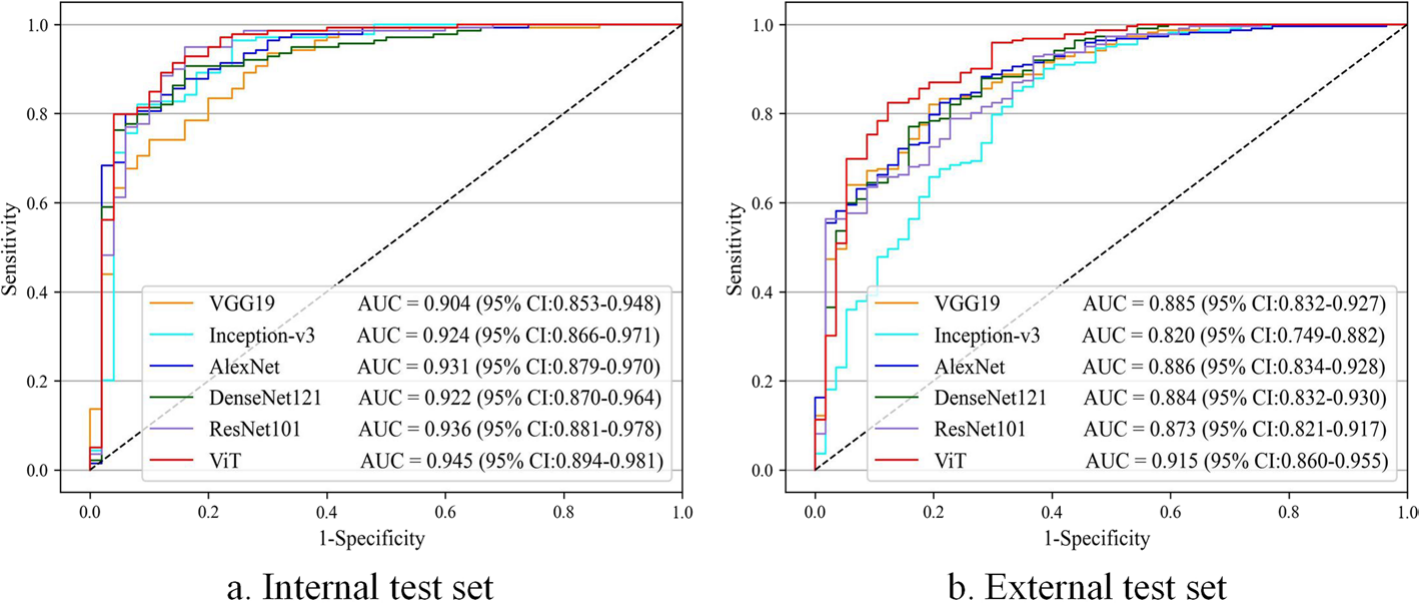
ROC curves of six deep learning algorithms in discerning malignant eyelid tumors. **a** ROC curves on the internal test set. **b** ROC curves on the external test set. *ROC* receiver operating characteristic, *AUC* area under the ROC curve, *CI* confidence interval

In this study, six deep learning algorithms were investigated to determine the optimal classifier for identifying benign and malignant eyelid tumors. Figure 5 displays the ROC curves and AUCs of these six algorithms in the classification of eyelid tumors. The ROC curve provides a comprehensive performance comparison of different models. The ROC curve of ViT was closer to the upper left, indicating its superior recognition performance. The optimal algorithm, ViT, achieved AUCs of 0.945 (95% CI 0.894–0.981) and 0.915 (95% CI 0.860–0.955) on the internal test set and external test set, respectively. Among the six algorithms, ResNet101 and Alexnet exhibited suboptimal performance on the internal and external test sets, with AUCs of 0.936 (95% CI 0.881–0.978) and 0.886 (95% CI 0.834–0.928), respectively. Compared with the suboptimal algorithms, the optimal algorithm of ViT improved the AUC by 0.009 and 0.029, respectively.

**Fig. 6:**
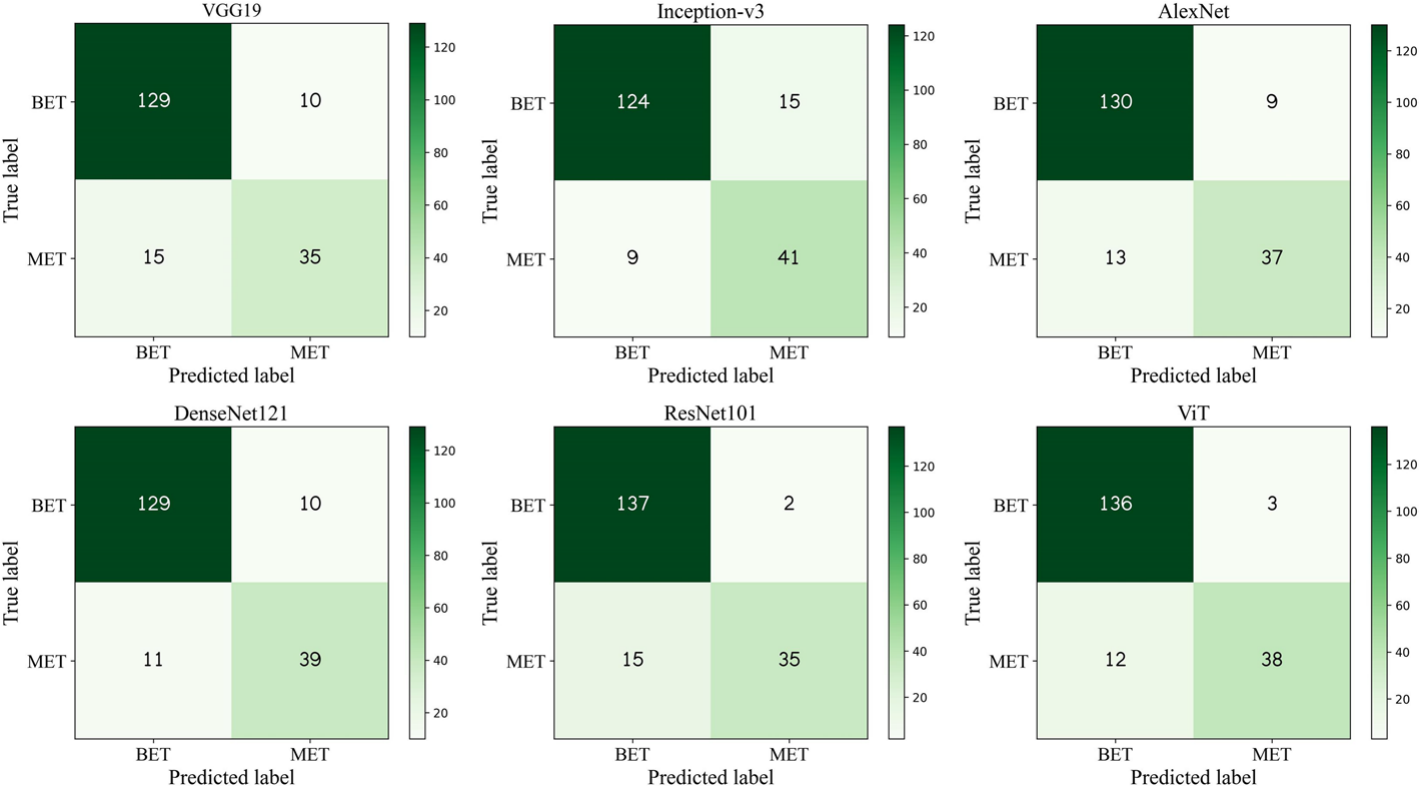
Confusion matrices of six deep learning algorithms on the internal test set

To analyze the specific number of correct and incorrect identifications of benign and malignant eyelid tumors, we presented the confusion matrices of the six deep learning algorithms on both the internal and external test sets. Experiment results were depicted in Fig. 6 and Additional file 1: Figure S1. ViT achieved the optimal recognition performance for both benign and malignant eyelid tumors on the internal and external test sets. It is noteworthy that ViT only misclassified three benign eyelid tumors while accurately identifying more malignant eyelid tumors. In contrast, on the internal test set, ResNet101 obtained better recognition performance only for benign eyelid tumors, and Inception-v3 showed better recognition performance only for malignant.

To comprehensively quantify the diagnostic performance of different deep learning algorithms for eyelid tumors, we calculated a variety of evaluation metrics, including sensitivity, specificity, accuracy, precision, and F1. Additional file 1: Table S1 provides a detailed comparison of experiment results. On the internal test set, the ViT algorithm achieved the highest accuracy and F1 of 92.1% (95% CI 88.2–95.9) and 83.5% (95% CI 78.3–88.8), respectively. In contrast, the Inception-v3 algorithm demonstrated the highest sensitivity of 82.0% (95% CI 75.6–88.4), while ResNet101 achieved the highest specificity of 98.6% (95% CI 95.3–100) and the best precision of 94.6% (95% CI 87.3–100). On the external test set, the ViT algorithm outperformed all other models in all quantitative metrics, achieving a sensitivity of 70.2% (95% CI 64.2–76.2), a specificity of 98.6% (95% CI 95.7–100), an accuracy of 92.8% (95% CI 89.8–95.9), a precision of 93.0% (95% CI 85.4–100), and an F1 of 80.0% (95% CI 75.3–84.7). The suboptimal ResNet101 algorithm achieved accuracies of 91.0% (95% CI 86.9–95.1) and 86.0% (95% CI 82.0–90.1) on the internal and external test sets, respectively. Compared with ResNet101, the ViT algorithm improved accuracies by 1.1% and 6.8% on the internal and external test sets, respectively.

**Fig. 7:**
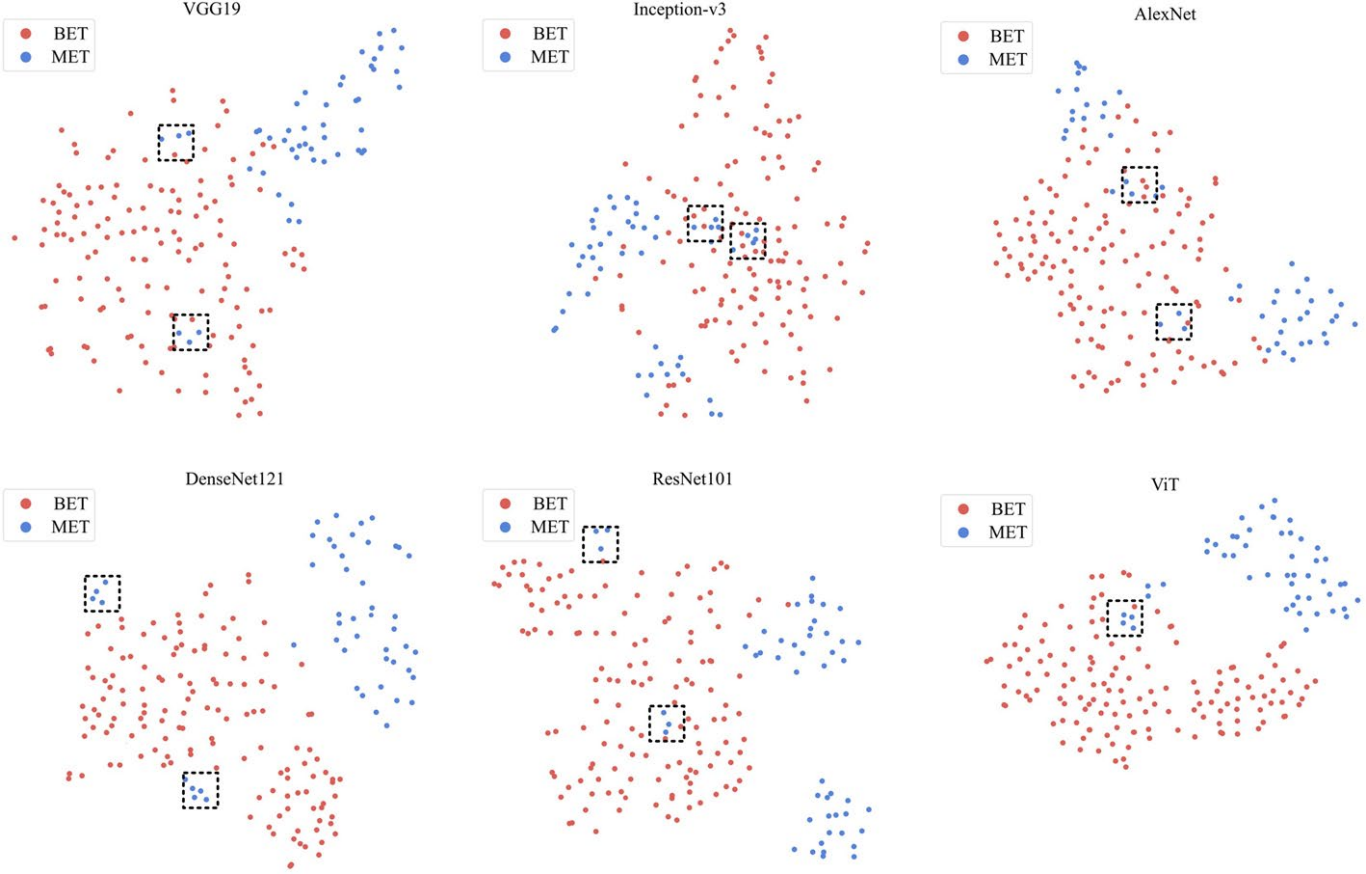
Visualization of the separability for the high-level features extracted by the six deep learning algorithms on the internal test dataset using t-SNE. The black-dotted rectangular box marks some of the indistinguishable samples. *t-SNE* t-distributed stochastic neighbor embedding

The t-distributed stochastic neighbor embedding (t-SNE) [28] technique was also applied to investigate whether the extracted high-level features are discriminative. The two-dimensional mapping of the high-level features enabled us to evaluate their ability to distinguish between benign and malignant eyelid tumors [29]. The t-SNE analysis revealed that the ViT achieved the best separability compared to other methods for embedding features in benign and malignant eyelid tumors (Fig. 7 and Additional file 1: Figure S2). However, it should be noted that the limited number of collected malignant eyelid tumor samples may have somewhat affected the separability of certain malignant cases.

**Fig. 8:**
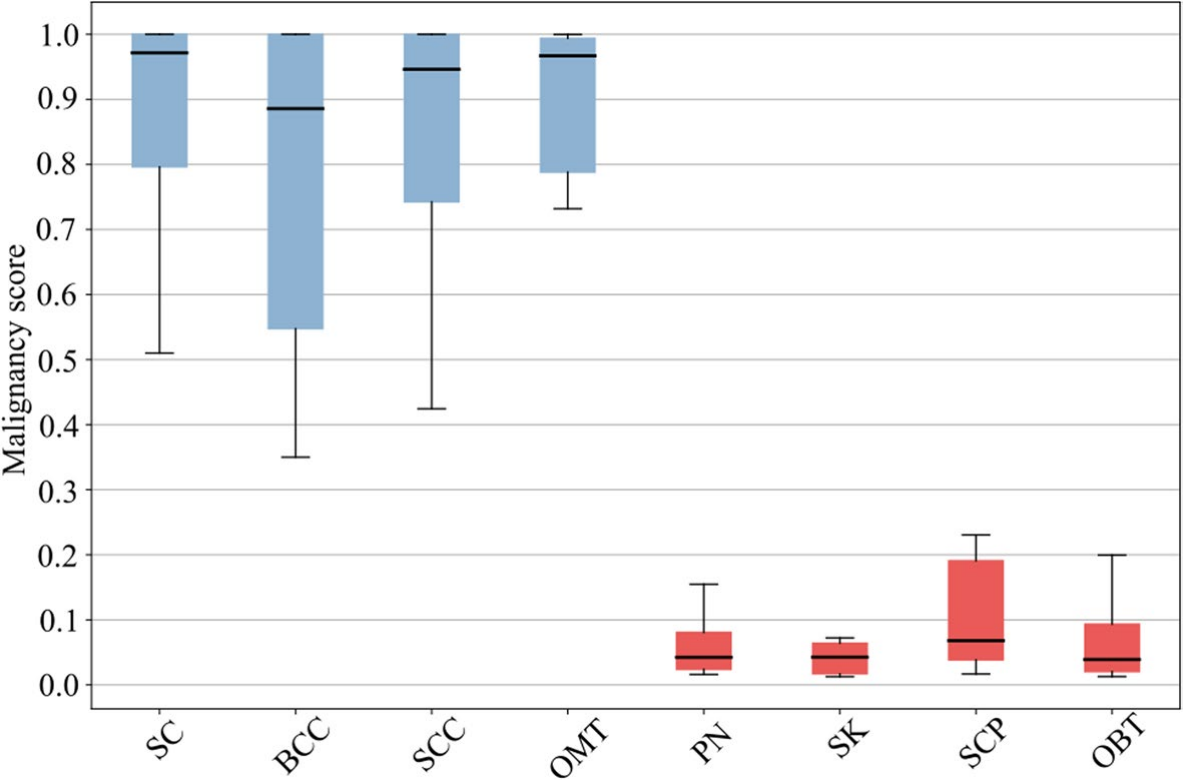
Malignancy scores (range 0–1) predicted by ViT algorithm for major categories of both malignant and benign eyelid tumors. The upper and lower boundaries of the box indicate the upper and lower quartiles of all scores, respectively. The intersection of the lines inside the box represents the median score

Malignant and benign eyelid tumors were labeled as 1 and 0, respectively. Figure 8 further displays the distribution of malignancy scores when identifying eyelid tumors using the optimal algorithm ViT. The ViT achieved high probability in correctly classifying malignant eyelid tumors, while its probability of misclassifying benign eyelid tumors as malignant was low. These results demonstrated that ViT achieved a high recognition rate in distinguishing between benign and malignant eyelid tumors. However, the misclassification rate of basal cell carcinomas (BCCs) was slightly higher, possibly due to their small lesion size, unclear boundaries, and morphological similarity to seborrheic keratosis.

#### Heatmap interpretability analysis of eyelid tumors

**Fig. 9:**
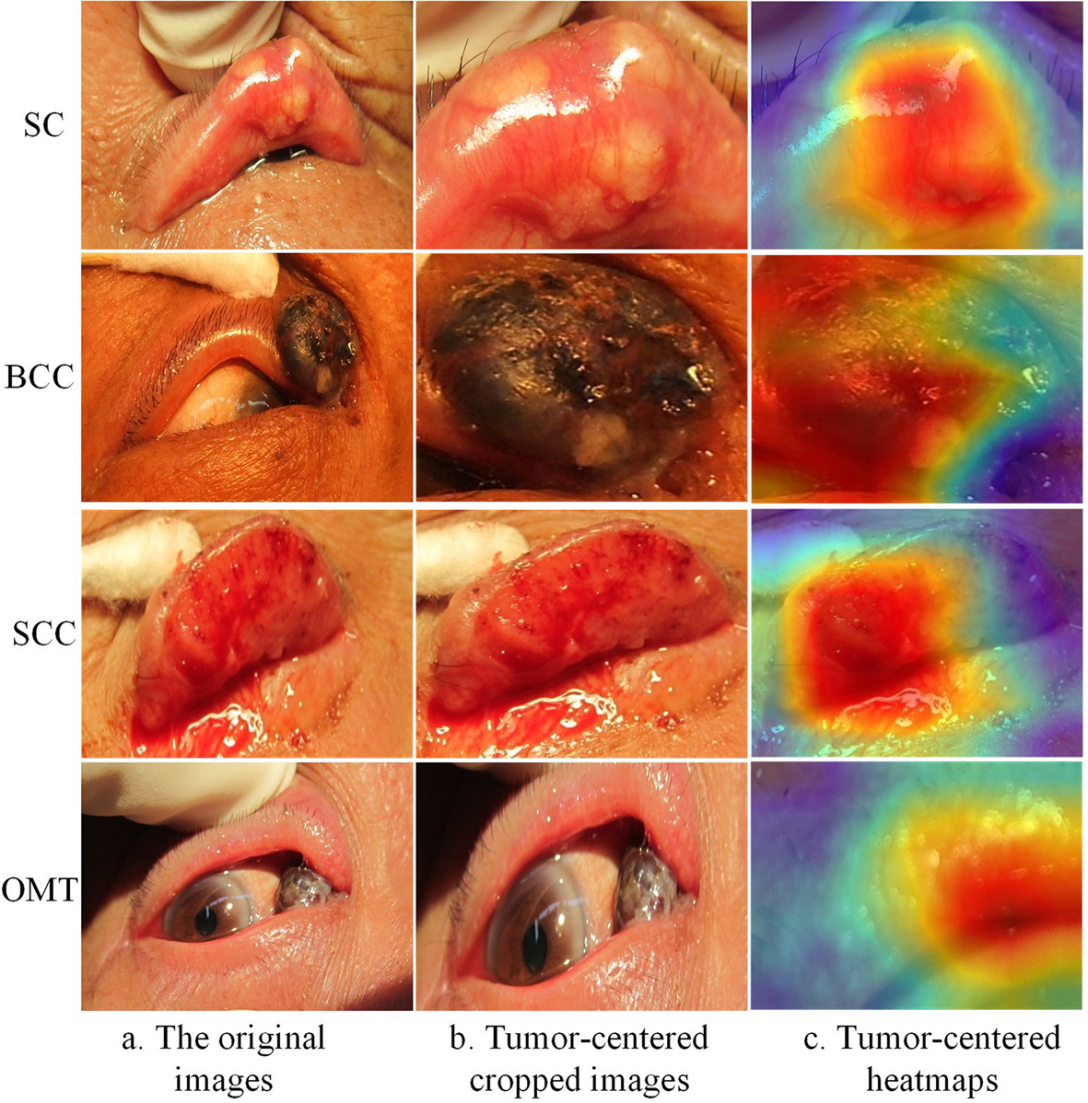
Representative examples of original images, cropped images, and corresponding heatmaps of malignant eyelid tumors

To investigate the interpretability of the ViT in classifying benign and malignant eyelid tumors, we utilized the gradient-weighted class activation mapping (Grad-CAM) [30] technique to generate heatmaps that visualized the regions contributing most to the model’s decisions. Typical original images, cropped images, and heatmaps of eyelid tumors are displayed in Fig. 9 and Additional file 1: Figure S3, respectively. From the experimental results, we can draw two meaningful conclusions. First, heatmaps accurately highlighted the lesion regions of both benign and malignant eyelid tumors, covering a variety of sizes, locations, and shapes. Second, the redder regions on the heatmaps represented more significant features identified using the ViT method. Therefore, the heatmaps results revealed that the critical lesions regions for identifying eyelid tumors, regardless of malignant or benign categories, could be accurately captured by the ViT.

## Discussion

In this study, a hybrid model HM_ADET was proposed for automatic diagnosis of eyelid tumors; in specific, YOLOv7_CNFG was used to automatically locate eyelid tumors and ViT was used for classification of benign and malignant. An efficient ConvNeXt module was explored in the YOLOv7_CNFG network to extract informative features of small lesions. Detailed comparative experiments represented that the YOLOv7_CNFG algorithm can effectively locate the regions of eyelid tumors in images. Moreover, the ViT classification network outperformed other conventional CNNs on both internal and external test datasets, which provided strong evidence of its superior performance and generalization ability. In addition, the Grad-CAM technique provided an interpretable pathway for the classification of benign and malignant eyelid tumors.

The YOLOv7_CNFG outperformed the conventional YOLOv7, mainly due to several factors. First, the dataset of eyelid tumor was preprocessed using mosaic data augmentation, and an adaptive anchor frame screening method was used to determine the size of anchor box. Second, the ConvNeXt network is employed to extract lesion features of small eyelid tumors, and its unique inverted bottleneck layer prevented information loss of small eyelid tumors during downsampling. Third, the FReLU activation function, designed specifically for visual tasks, captures a detailed features of eyelid tumor images such as texture, edges, and shapes, improving the accuracy of object localization. In addition, due to the complex shape and irregular boundaries of eyelid tumors, the GIoU_Loss is used to calculate the boundary loss of the object box. The localization boxes of YOLOv7_CNFG model almost perfectly matched the true boxes labeled by the ophthalmologist. Compared with YOLOv7, YOLOv7_CNFG achieved improvements of 0.13 and 0.118 in AP on the internal and external test sets, respectively.

Compared with previous studies, our proposed method for automatically diagnosing eyelid tumors showed satisfactory results. First, the eyelid tumor was relatively small and surrounded by noise. The YOLOv7_CNFG localization method was employed to locate the eyelid tumor prior to diagnosis, preventing the noise being transmitted to the classifier to affect the diagnosis performance. Second, early-stage benign and malignant eyelid tumors often present similar lesion characteristics. ViT algorithm was adopt to self-attention mechanism and MLP to achieve benign and malignant classification. Experimental results indicated that the ViT algorithm achieved better performance, with AUCs of 0.945 (95% CI 0.894–0.981) and 0.915 (95% CI 0.860–0.955) on the internal and external test sets for identifying malignant eyelid tumors, respectively. When compared to the suboptimal algorithm ResNet101, ViT improved the sensitivity and accuracy by 6.0% and 1.1% on the internal test set and by 12.3% and 6.8% on the external test set.

The impact of automatic localization results on the classification model was further analyzed. For the internal test dataset, there were a total of 189 eyelid tumor regions, out of which 169 were successfully located, indicating that automatic localization model performed well. Tumors that were not located unavoidably had an impact on the performance of the entire diagnostic system. This impact potentially reduced the accuracy of the entire diagnostic system by approximately 10%. However, the high-precision localization model itself held significant clinical value. It substantially facilitated ophthalmologists in rapidly identifying the suspicious locations of eyelid tumors from captured images, regardless of whether they were benign or malignant. On the other hand, the classification model, by distinguishing between benign and malignant tumors, also provided diagnostic guidance for ophthalmologists and patients. This aligned with the evidence-based diagnostic approach for eyelid tumors in clinical practice.

To demonstrate the interpretability of ViT, the Grad-CAM technique was applied to generate heatmaps to visualize the regions of interest in the localization of eyelid tumors. The lesion regions were identified as critical areas in the images, providing further evidence of the model’s efficacy. In specific, eight heatmaps of typical benign and malignant eyelid tumors were presented, accurately highlighting the lesion regions. The closer to the center of the lesion, the more intense the red color appears, indicating that the model is paying more attention to this part of the region. This interpretable exploration significantly facilitates the practical clinical application of ViT.

Due to its reliable performance, our method can be utilized in large-scale screening and disease confirmation stage after the consultation, facilitating the early identification of malignant eyelid tumors. In regions with poor medical infrastructure, early screening is critical to ensure patients receive timely treatment. Distinguishing between malignant and benign eyelid tumors is challenging for junior ophthalmologists because of the small size, uneven location distribution, and the structural similarity of these tumors. Our automatic diagnostic method can aid ophthalmologists in the efficient diagnosis of patients with eyelid tumors. Therefore, it not only improves the diagnostic efficiency of ophthalmologists, but also reduces medical costs by avoiding unnecessary examination of apparently benign eyelid tumors.

Our study has several limitations. First, the number of collected images of eyelid tumors is relatively small, which makes it challenging to study finer classification. In particular, the limited number of malignant eyelid tumors makes it difficult to distinguish between its subtypes. Second, although our method provides a practical strategy for the automatic diagnosis of benign and malignant eyelid tumors, its sensitivity is slightly lower due to the similar phenotypes between these two types of eyelid tumors. Furthermore, while our algorithm is well-suited for screening purpose, it is not suitable for providing a specific subtype diagnosis based solely on images. Combining electronic medical records and other optical images, multimodal fusion algorithms will be explored to provide valuable supplements for the comprehensive assessment of eyelid tumors.

## Conclusions

In this study, we propose a hybrid model HM_ADET for automatically diagnosis of eyelid tumors. This model includes YOLOv7_CNFG for automatic localization of small eyelid tumors and ViT classification algorithm. YOLOv7_CNFG integrates ConvNeXt pure convolutional network, FReLU visual activation function, and GIoU_loss function. The YOLOv7_CNFG has a high localization performance on both internal and external test sets, making it a promising tool for clinical diagnosis of eyelid tumors. The experimental results and comparative analysis verify that the ViT classification algorithm outperforms other CNNs in identifying benign and malignant eyelid tumors. Interpretability experiments and external test set validation indicate that YOLOv7_CNFG object localization algorithm and ViT classification algorithm have excellent rationality and generalization ability in clinical applications. Overall, our study provides a valuable reference for the automatic diagnosis of not only eyelid tumors but also other eye diseases.

## Methods

### Overview architecture of the automatic detection of eyelid tumors

**Fig. 10:**
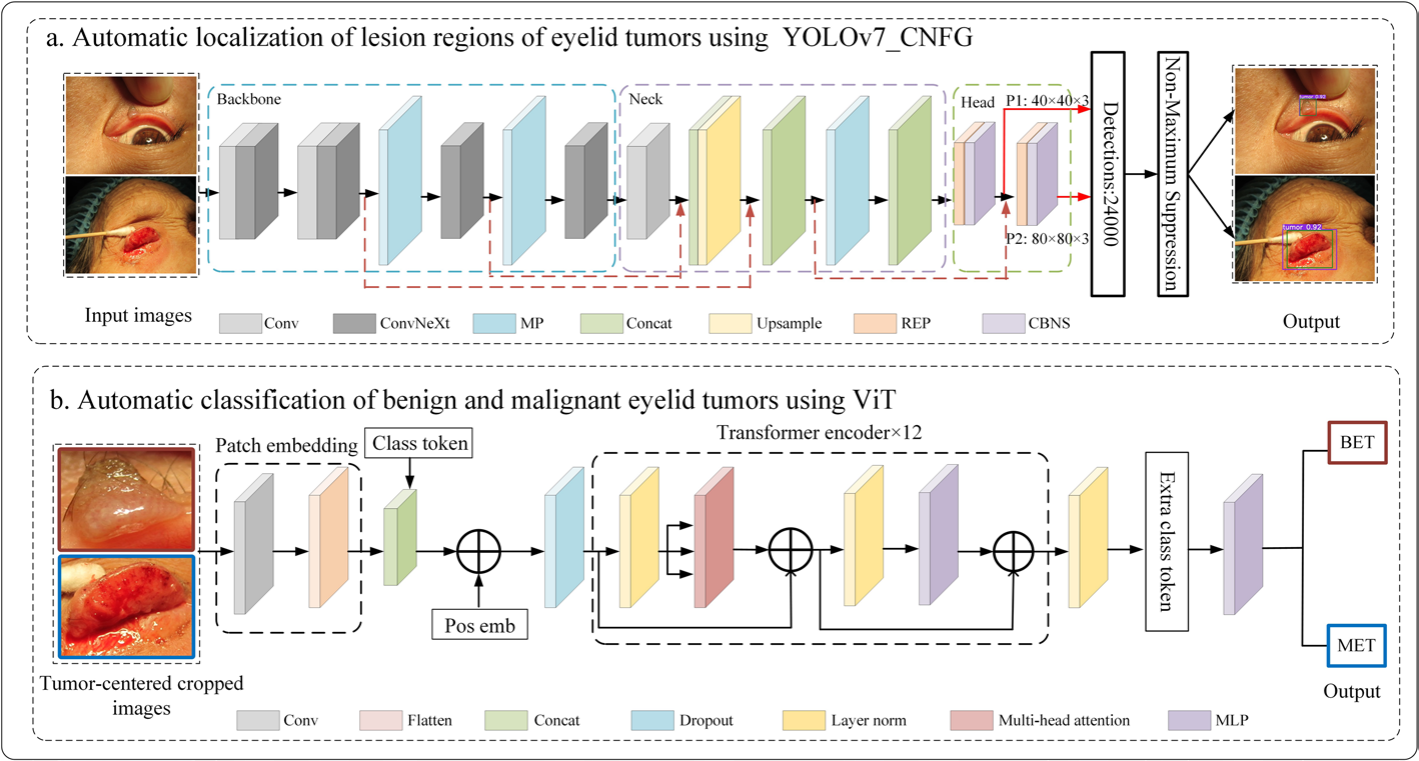
The HM_ADET framework of automatic detection of eyelid tumors. *ViT* vision transformer, *YOLOv7_CNFG* YOLOv7 + ConvNeXt + FReLU + GIoU_loss, *BET* benign eyelid tumors, *MET* malignant eyelid tumors

As depicted in Fig. 10, the HM_ADET architecture of the automatic detection of eyelid tumors consists of two stages: automatic localization of lesion regions of eyelid tumors (Fig. 10a) and automatic classification of benign and malignant eyelid tumors (Fig. 10b). In Fig. 10a, the automatic localization algorithm YOLOv7_CNFG consists of four modules: input, backbone, neck, and head. The mosaic data augmentation technique is utilized in the input module to enhance dataset diversity. The backbone module is a crucial functional component responsible for performing convolution, normalization, and pooling operations on input images. ConvNeXt, a pure convolutional network [31], is employed in the backbone module to extract distinctive features from input images, achieving better accuracy than swim transformer with similar computational overhead [32, 33]. The neck module can accurately locate eyelid tumor lesions by integrating multi-scale features. The output module is used to output the located category and the localization boundary. We extracted two shallow features from the ConvNeXt module and fused them to obtain the output feature maps, P1 and P2, with sizes of 40 × 40 and 80 × 80, respectively, suitable for locating medium and small eyelid tumors. In Fig. 10b, the ViT model is employed for automatic classification of benign and malignant eyelid tumors. ViT converts the located image data into the input form required by transformer using the patch embedding module. After stacking a series of transformer encoder blocks, a multilayer perceptron (MLP) directly classifies benign and malignant eyelid tumors. In the following sections, the functions and novelty of each module, the adopted activation function, and the loss function are introduced in detail.

### ConvNeXt network

**Fig. 11:**
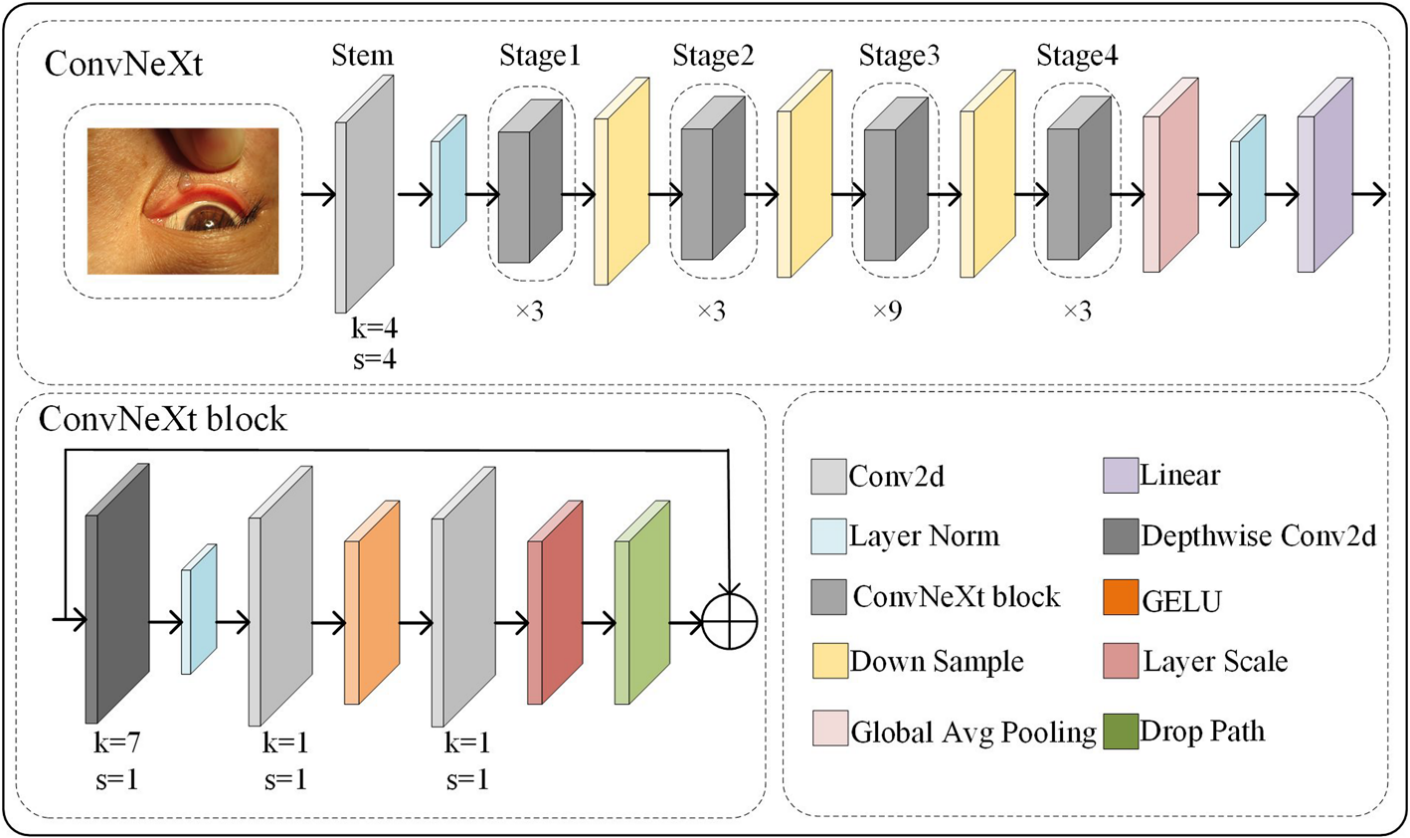
The network structure of the ConvNeXt and its block. The “ × 3, × 3, × 9, and × 3” indicate that stages 1, 2, 3, and 4 have 3, 3, 9, and 3 blocks, respectively

With the advancement of deep learning algorithms, the swin transformer has gained attention as a promising alternative to CNNs for object localization applications due to its superior performance in various benchmarks. The ConvNeXt network was proposed by building upon the swin transformer and leveraging its layer structure, downsampling, activation function, inverse bottleneck, and deep convolution to further enhance the localization accuracy [33]. ConvNeXt possesses powerful feature extraction capabilities while maintaining low hardware requirements. Due to the small lesion regions of eyelid tumors, the downsampling operation in the YOLOv7 easily lose the features of small eyelid tumors. Therefore, this study adopts the ConvNeXt to address the problem of insufficient features extraction of small eyelid tumors. The network structure of ConvNeXt and its block is shown in Fig. 11.

The ConvNext network comprises a stem and four stages, with each stage containing a different number of blocks in a ratio of 3:3:9:3. To adjust the number of channels, ConvNeXt network performs downsampling operation using a convolutional layer with a kernel size equal to its stride. After global average pooling and layer normalization (LN) operation, a linear layer produces the downsampling result. Batch normalization is commonly used for LN operation to stabilize models and reduce gradient oscillation. Given the significant correlation between the input of a layer and the output of the previous layer, LN can address the issue of covariate shift by specifying the mean (µ) and variance (σ) of the input summation for each layer [34]. In specific, the statistical mean and variance of LN for all hidden units within a given layer can be calculated using formulas (5–6).

5 $$\mu^{l} = \frac{1}{{\text{H}}}\sum\limits_{i = 1}^{{\text{H}}} {a_{i}^{l} } \sigma^{l}$$

6 $$\sigma^{l} = \sqrt {\frac{1}{{\text{H}}}\sum\limits_{i = 1}^{{\text{H}}} {(a_{i}^{l} } - \mu^{l} )^{2} }$$

where H refers to the number of hidden units within a given layer, $l$ represents the $l{{{\text{th}}}}$ hidden layer, and $a^{l}$ denotes the summed input vector to the neurons in this layer. $a_{i}^{l}$ is the summed input to the $i$th hidden unit in the $l$th layer.

In each stage of the ConvNeXt network, a series of ConvNeXt blocks are used to extract features. Each block contains a branch that performs depth-wise separable convolution with a size of 7 × 7, balancing accuracy and computational overhead. The convolution between channels involves the correlation between channels, while the convolution between spaces focuses on the spatial location correlation. This separable convolution makes the convolution kernel more focused on learning specific feature information, reducing information confusion and loss. Then, a 1 × 1 convolution layer is applied to increase the number of channels by four times, followed by downsampling through another 1 × 1 convolution layer to restore the original number of channels, minimizing the loss of high-dimensional information. Finally, the scale layer scales the data of each channel, and the drop path layer implements random dropout of neurons to prevent the model from converging to a local optimal solution caused by noisy data. Through the operations of dimension-up convolution and dimension-reduction convolution, the inverse bottleneck layer structure can improve the model’s expressiveness and feature extraction ability while minimizing information loss, and retain the key information in the input feature map, thus improving the accuracy and robustness of the model.

### FReLU activation function

Various convolutional techniques, such as dilated and deformable convolutions, have been proposed to capture the spatial relationships of features and adaptively incorporate local contextual information in images [35]. However, such techniques often increase network complexity. To overcome this challenge, we replace these convolutions with the FReLU function, a funnel-shaped activation function specifically designed for visual tasks. The FReLU utilizes the same operation as the $\max ( \cdot )$ nonlinear function to capture spatial relationships. It also unfolds the conditional part to a two-dimensional condition that depends on the spatial context for each pixel. The specific expression of FReLU is shown in Eq. (7).

7 $$f(x_{c,i,j} ) = \max (x_{c,i,j} ,T(x_{c,i,j} ))$$

where $x_{c,i,j}$ represents the input pixel of the nonlinear activation function $f( \cdot )$ at the two-dimensional space position $(i,j)$ in the C channel. $T(x_{c,i,j} )$ is a two-dimensional condition that leverages a parametric pooling window to create spatial dependencies, as described by Eq. (8).

8 $$T(x_{c,i,j} ) = x_{c,i,j}^{w} \cdot p_{c}^{w} $$

where $x_{c,i,j}^{w}$ denotes a $k_{h} \times k_{w}$ parametric pooling window centered at $x_{c,i,j}$, $p_{c}^{w}$ denotes the coefficient shared in the same channel on this window. $( \cdot )$ denotes dot product.

Using the funnel condition, the network is able to generate spatial conditions for the nonlinear activation of each pixel. The network can perform nonlinear transformations and generate spatial dependencies in the convolutional layers simultaneously. In addition, the per-pixel condition enables the network with the ability to model each pixel individually, and the function $\max ( \cdot )$ provides a choice whether to consider spatial context for each pixel. The nonlinearity of FReLU can better capture and express complex and abstract features in images. In object localization tasks, locating small objects often requires the use of multi-scale features to improve accuracy, and the nonlinear properties can help neural networks utilize feature information at different scales [36, 37]. Therefore, the implementation of this technique effectively improves the localization accuracy of small eyelid tumors.

### GIoU_loss function

The YOLOv7 network utilizes the complete intersection over union (CIoU) metric to calculate the loss of boundary box regression [38]. However, due to the diverse shapes and boundaries of eyelid tumors, locating small eyelid tumors can be especially difficult. Moreover, the CIoU_loss does not fully consider the issue of PB and GT not intersecting. To address these issues, the generalized intersection over union (GIoU) is introduced as a novel loss for object localization [39]. The GIoU_loss function considers the geometric center differences between the PB and GT, which can better handle complex shapes and irregular boundaries, thereby improving the accuracy of eyelid tumor localization. When PB and GT do not overlap, the GIoU_loss function can consider the overlap of the PB and GT based on the area of their bounding rectangles. This approach ensures that the model can be trained normally and learn useful information even when the PB and GT do not intersect.

**Fig. 12:**
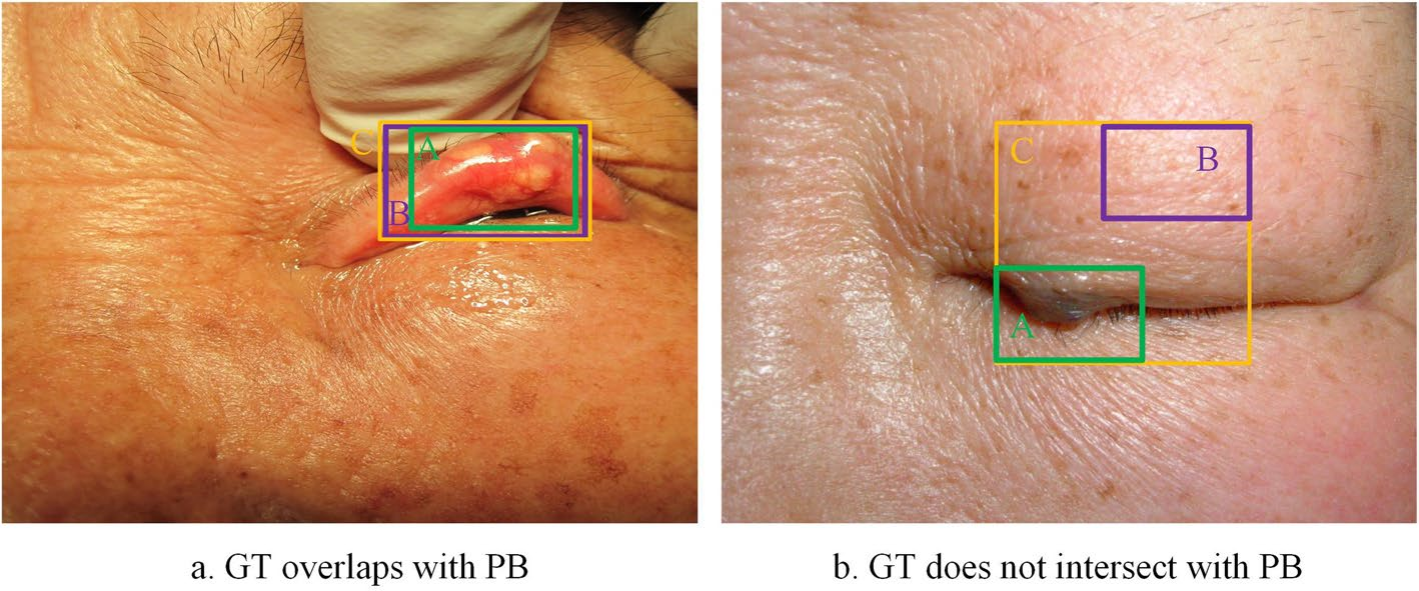
The schematic diagram of GIoU. The green box A refers to the area of GT. The purple box B refers to the area of PB. The orange box C refers to the minimum bounding rectangle that contains A and B. *GIoU* generalized intersection over union, *GT* ground truth box, *PB* predicted box

Figure 12 illustrates the concept of GIoU. GIoU is defined as any two boxes (A, B) in the set S of all boxes. First, it is necessary to find the minimum bounding rectangle C that enclose both A and B, and calculate the absolute value of the ratio of C to the union of A and B, that is $\left| {{\text{C}}\backslash ({\text{A}} \cup {\text{B}})} \right|$, to obtain the differing part between the two. Then, calculate the ratio of $\left| {{\text{C}}\backslash ({\text{A}} \cup {\text{B}})} \right|$ to the absolute value of C. Finally, subtract the calculated ratio from the intersection over union (IOU) values of A and B to obtain the GIoU. The size of ${\text{C}}\backslash ({\text{A}} \cup {\text{B}})$ is closely related to the distance between A and B. Therefore, GIoU expresses the distance between PB and GT [40], which is more conducive to features learning during training process and enables PB to be closer to GT.

The formula for calculating GIoU_Loss is presented in Table 4, which measures the intersection ratio of PB and GT. The application of GIoU can address the problem of loss function lacking gradient when PB and GT do not intersect [41]. In the context of eyelid tumor localization, GIoU_loss can effectively reduce the rate of missed and false localizations of small eyelid tumors.

**Table 4:**
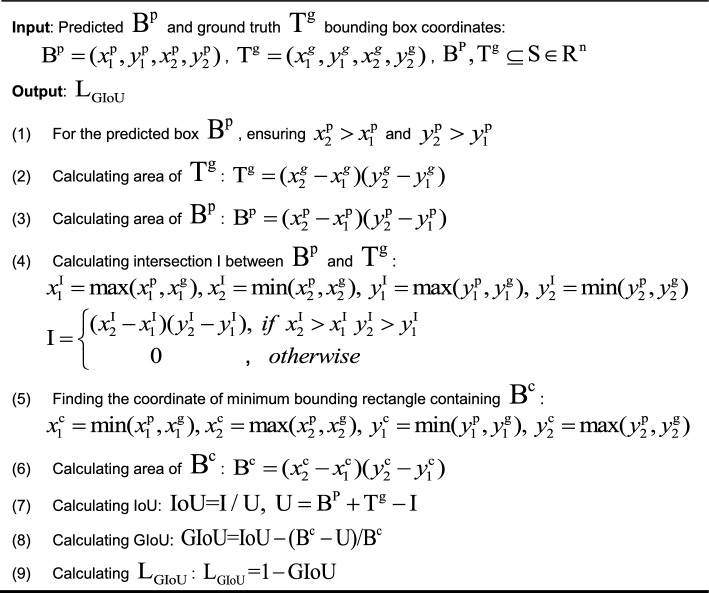
Algorithmic formula of GIoU_loss

$(x_{1} ,y_{1} )$ and $(x_{2} ,y_{2} )$ represent the coordinates of the top-left corner and bottom-right corner of the bounding box, respectively

### Supplementary Information

**Additional file 1: Figure S1.** Confusion matrices of six deep learning algorithms on the external test. **Figure S2.** Visualization of the separability for the high-level features extracted by the six deep learning algorithms on the external test dataset using t-SNE. The black dotted rectangular box marks some of the indistinguishable samples.t-SNE, t-distributed stochastic neighbor embedding. **Figure S3.** Representative examples of original images, cropped images and corresponding heatmaps of benign eyelid tumors. **Table S1.** Performance comparison of six deep learning algorithms for identifying eyelid tumors on both internal and external test sets.

## Data Availability

The source code and example data used in this study can be accessed at GitHub (https://github.com/jiangjiewei/Eyelid-tumors-HM_ADET). The data generated and/or analyzed during the study are available upon reasonable request from the corresponding author. Correspondence and requests for data materials should be addressed to Zhongwen Li.

## Abbreviations

FReLU: Flexible rectified linear unit
PB: Predicted box
GT: Ground truth box
MHA: Multi-head attention
DL: Deep learning
DR: Diabetic retinopathy
CNNs: Convolutional neural networks
DBNs: Deep belief networks
ELM: Extreme learning machine
GIoU_loss: Generalized intersection over union loss
NEH: Ningbo Eye Hospital
JEH: Jiangdong Eye Hospital
ZFPH: Zunyi First People’s Hospital
BET: Benign eyelid tumors
PN: Pimented naevus
SK: Seborrheic keratosis
SCP: Squamous cell papilloma
OBT: Other benign tumor
MET: Malignant eyelid tumors
SC: Sebaceous carcinoma
BCC: Basal cell carcinoma
SCC: Squamous cell carcinoma
OMT: Other malignant tumor
AP: Average precision
GFLOPs: Giga floating-point operations per second
PR: Precision–recall
TP: True positive
FP: False positive
FN: False negative
F1: F1-score
ROC: Receiver operating characteristic
AUC: Area under the ROC curve
CI: Confidence intervals
t-SNE: T-distributed stochastic neighbor embedding
Grad-CAM: Gradient-weighted class activation mapping
CIoU: Complete intersection over union
GIoU: Generalized intersection over union
