# Supplementary material for: HM_ADET: a hybrid model for automatic detection of eyelid tumors based on photographic images
Source: Biomed Eng Online. 2024 Feb 28;23:25. doi: 10.1186/s12938-024-01221-3 (PMC10903075; doi:10.1186/s12938-024-01221-3)
Supplement: Supplementary file 1 — Additional file 1: Figure S1. Confusion matrices of six deep learning algorithms on the external test. Figure S2. Visualization of the separability for the high-level features extracted by the six deep learning algorithms on the external test dataset using t-SNE. The black dotted rectangular box marks some of the indistinguishable samples.t-SNE, t-distributed stochastic neighbor embedding. Figure S3. Representative examples of original images, cropped images and corresponding heatmaps of benign eyelid tumors. Table S1. Performance comparison of six deep learning algorithms for identifying eyelid tumors on both internal and external test sets. [file 12938_2024_1221_MOESM1_ESM.docx]

**Title:** **HM_ADET: A Hybrid Model for Automatic Detection of Eyelid Tumors Based on Photographic Images**

**Additional Information**

1. **Additional Fig 1.** Confusion matrices of six deep learning algorithms on the external test.
2. **Additional Fig 2.** Visualization of the separability for the high-level features extracted by the six deep learning algorithms on the external test dataset using t-SNE. The black dotted rectangular box marks some of the indistinguishable samples.t-SNE, t-distributed stochastic neighbor embedding.
3. **Additional Fig 3.** Representative examples of original images, cropped images and corresponding heatmaps of benign eyelid tumors.
4. **Additional Table 1.** Performance comparison of six deep learning algorithms for identifying eyelid tumors on both internal and external test sets.

**
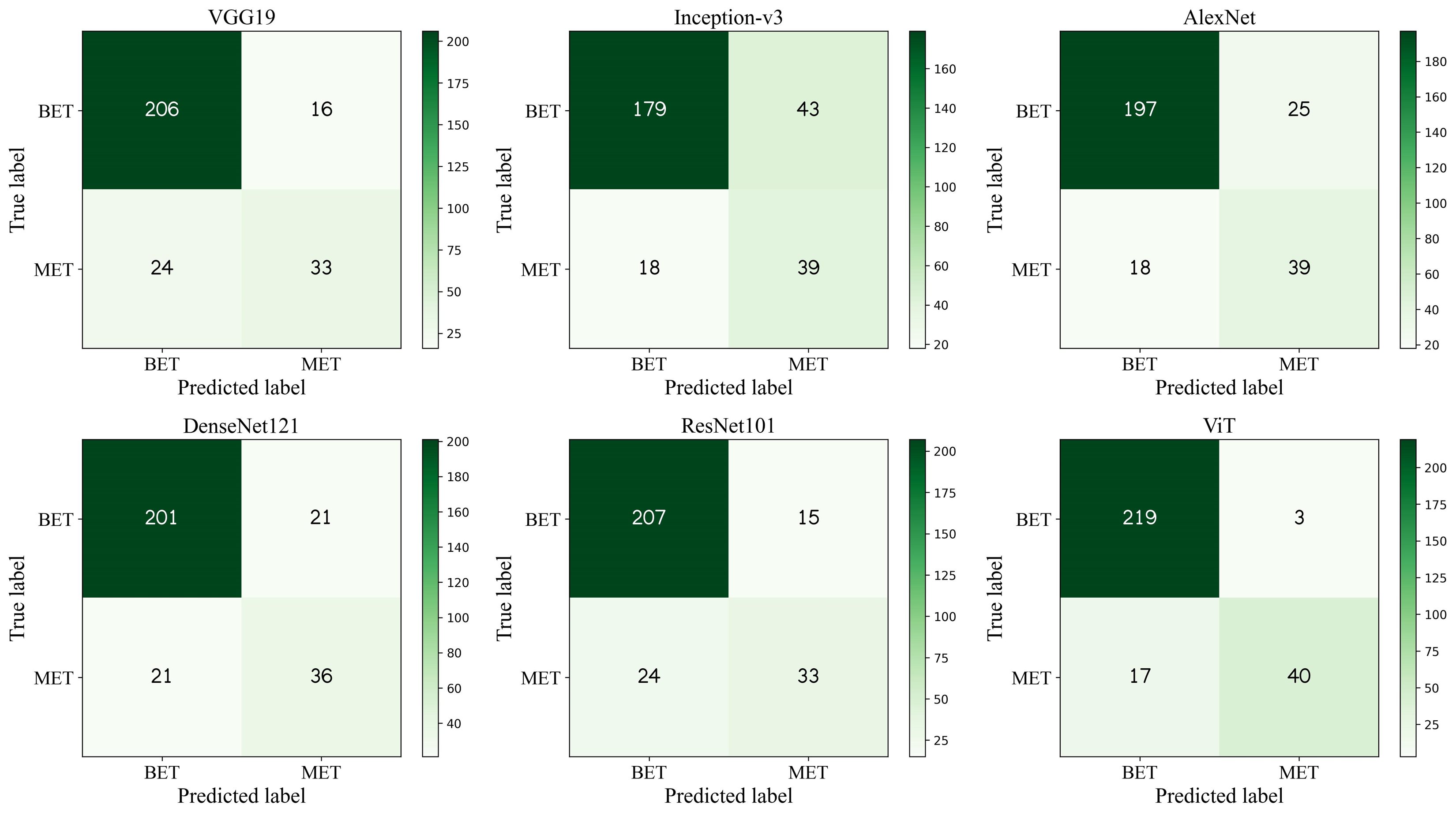
**

**Additional Fig 1.** Confusion matrices of six deep learning algorithms on the external test set.


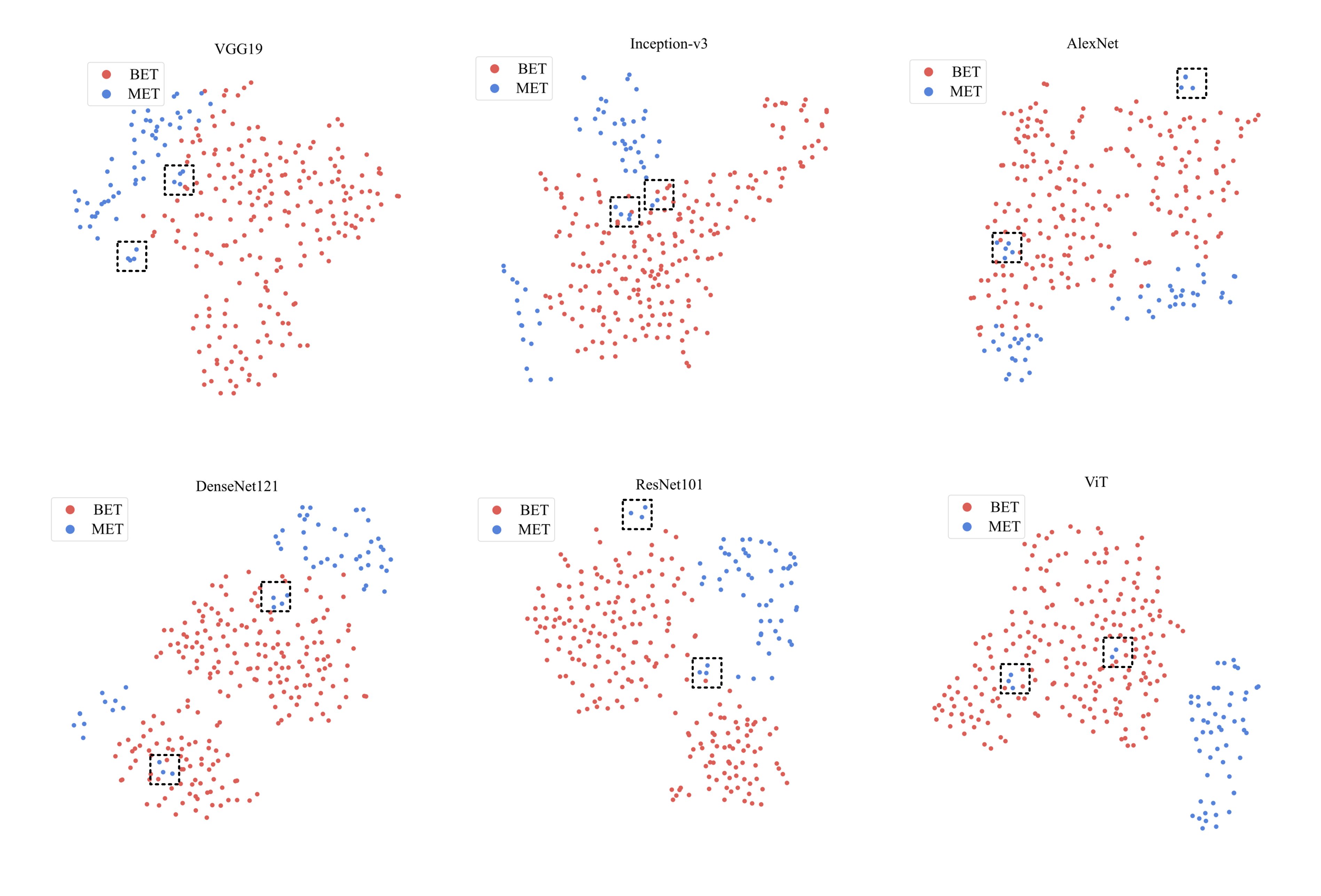


**Additional Fig 2.** Visualization of the separability for the high-level features extracted by the six deep learning algorithms on the external test dataset using t-SNE. The black dotted rectangular box marks some of the indistinguishable samples.t-SNE, t-distributed stochastic neighbor embedding.

**
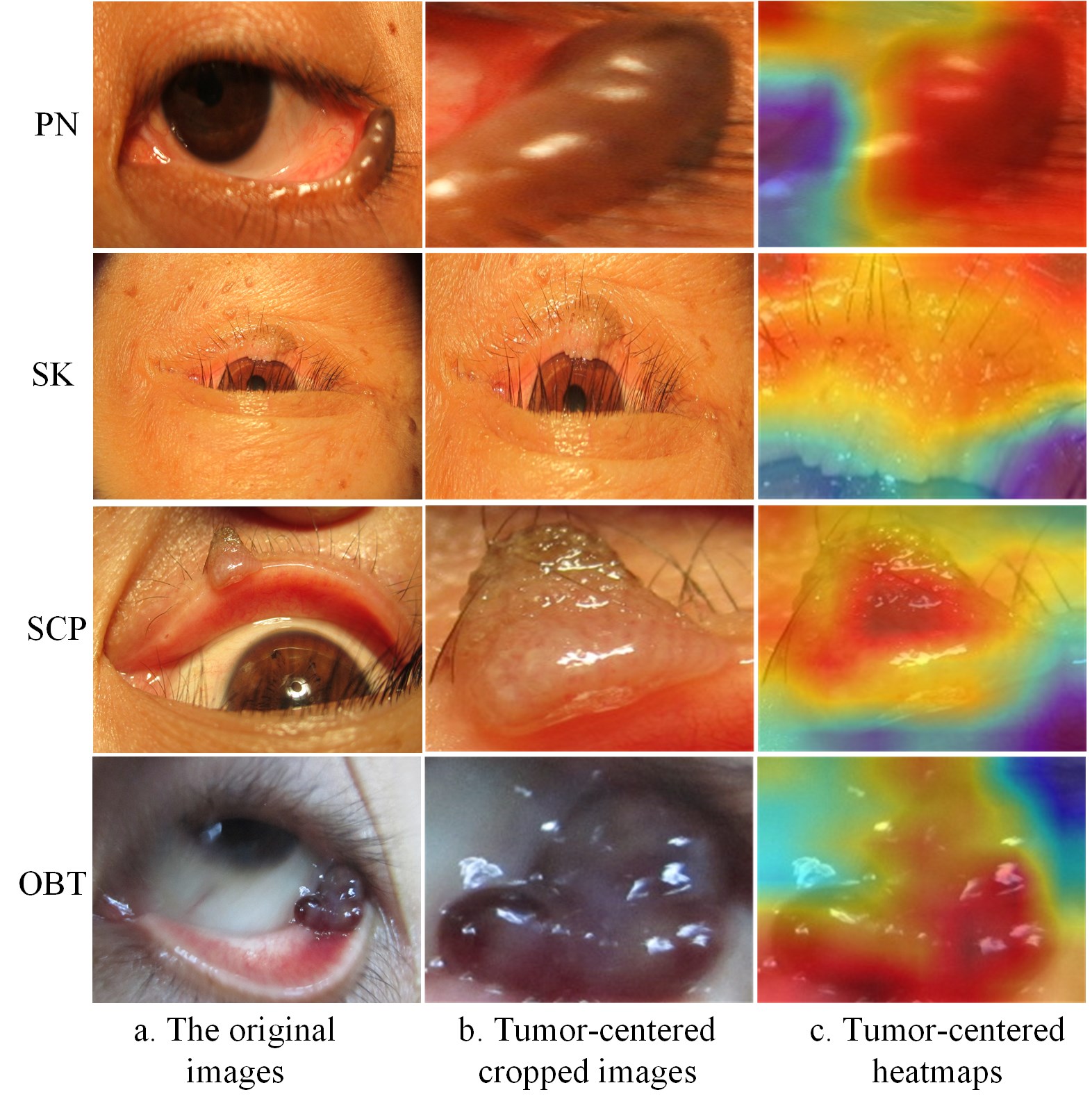
**

**Additional Fig 3.** Representative examples of original images, cropped images and corresponding heatmaps of benign eyelid tumors.

**Additional Table 1. Performance comparison of six deep learning algorithms for identifying eyelid tumors on both internal and external test sets.**

| **Algorithm** | **Test data** | **Sensitivity(95%CI)** | **Specificity(95%CI)** | **Accuracy(95%CI)** | **Precision(95%CI)** | **F1(95%CI)** |
| --- | --- | --- | --- | --- | --- | --- |
| VGG19 | Internal test data | 70.0%(62.4-77.6) | 92.8%(85.6-100) | 86.8%(81.9-91.6) | 77.8%(65.6-89.9) | 73.7%(67.4-80.0) |
|  | External test data | 57.9%(51.4-64.4) | 92.8%(86.1-99.5) | 85.7%(81.6-89.8) | 67.3%(54.2-80.5) | 62.3%(56.6-68.0) |
| Inception-v3 | Internal test data | **82.0%(75.6-88.4)** | 89.2%(80.6-97.8) | 87.3%(82.6-92.0) | 73.2%(61.6-84.8) | 77.4%(71.4-83.3) |
|  | External test data | 68.4%(62.3-74.5) | 80.6%(70.4-90.9) | 78.1%(73.3-83.0) | 47.6%(36.8-58.4) | 56.1%(50.3-61.9) |
| AlexNet | Internal test data | 74.0%(66.7-81.3) | 93.5%(86.7-100) | 88.4%(83.8-92.9) | 80.4%(69.0-91.9) | 77.1%(71.1-83.1) |
|  | External test data | 68.4%(62.3-74.5) | 88.7%(80.5-96.9) | 84.6%(80.4-88.8) | 60.9%(49.0-72.9) | 64.5%(58.8-70.1) |
| DenseNet121 | Internal test data | 78.0%(71.1-84.9) | 92.8%(85.6-99.9) | 88.9%(84.4-93.4) | 79.6%(68.3-90.9) | 78.8%(73.0-84.6) |
|  | External test data | 63.2%(56.8-69.5) | 90.5%(82.9-98.1) | 84.9%(80.8-89.1) | 63.2%(50.6-75.7) | 63.2%(57.5-68.8) |
| ResNet101 | Internal test data | 70.0%(62.4-77.6) | **98.6%(95.3-100)** | 91.0%(86.9-95.1) | **94.6%(87.3-100)** | 80.5%(74.8-86.1) |
|  | External test data | 57.9% (51.3-64.4) | 93.2% (86.7-99.8) | 86.0% (82.0-90.1) | 68.8%(55.6-81.9) | 62.9% (57.2-68.5) |
| ViT | Internal test data | 76.0% (68.9-83.1) | 97.8% (93.8-100) | **92.1%(88.2-95.9)** | 92.7% (84.7-100) | **83.5% (78.3-88.8)** |
|  | External test data | **70.2%(64.2-76.2)** | **98.6%(95.7-100)** | **92.8%(89.8-95.9)** | **93.0%(85.4-100)** | **80.0%(75.3-84.7)** |

**Footnote:** The bold font is the optimal value of each algorithm on the internal and external test sets.
